# Thyroid Predictors of Postpartum Mood Disorders

**DOI:** 10.7759/cureus.45554

**Published:** 2023-09-19

**Authors:** Sean Backer, Janeta Yancheva, Camelia Garcia, Deepesh Khanna

**Affiliations:** 1 Osteopathic Medicine, Dr. Kiran C. Patel College of Osteopathic Medicine, Nova Southeastern University, Tampa, USA; 2 Foundational Sciences, Dr. Kiran C. Patel College of Osteopathic Medicine, Nova Southeastern University, Clearwater, USA; 3 Foundational Sciences, Dr. Kiran C. Patel College of Osteopathic Medicine, Nova Southeastern University, Fort Lauderdale, USA

**Keywords:** second and third trimester, late postpartum depression, tpo-antibody positive, hypothyroidism, thyroid markers, postpartum psychosis, postpartum blues, postpartum depression, postpartum mood disorders, thyroid predictors

## Abstract

Postpartum mood disorders (PMD) are currently among the leading causes of maternal postpartum morbidity and mortality. PMD include the conditions of postpartum blues (PB), postpartum depression (PPD), and postpartum psychosis. The pathogenesis of PMDs are ambiguous, and there are no reliable prenatal predictive markers despite current research efforts. Even though reliable indicators have not been found, leading ideas suggest an etiology of hormonal fluctuations. Although thyroid markers have long been linked to psychiatric disorders such as major depressive disorder (MDD), how they correlate with PMDs is still unclear. This study aimed to evaluate the pathophysiological link between thyroid function, PMDs, and the usefulness of thyroid markers as indicators of their occurrence and severity.

The methodology consisted of a narrative literature review. Several inclusion and exclusion criteria were used to filter the results of literature searches in PubMed. Studies were included if they discussed any marker related to thyroid endocrinology in relation to the incidence or pathophysiology of any PMD. Both primary and secondary analyses were included. The permissive inclusion criteria were used due to the relative scarcity of research on the topic and the ambiguous pathophysiology of PMD.

The results demonstrated the potential utility of thyroid autoimmunity as a predictor of late-onset PPD. Hypothyroidism, low euthyroid hormone levels, and the presence of thyroid autoantibodies were correlated with increased incidence of PPD and late postpartum depressive symptoms, past the timeline of PB. Most notably a rapid postpartum drop in cortisol level may precipitate thyroid autoimmunity in anti-thyroid peroxidase (TPO) antibody positive women, which could eventually produce a hypothyroid phase associated with depressive symptoms. There was insufficient evidence to suggest a relationship with postpartum psychosis. In conclusion, the exact pathophysiological mechanisms of PMDs remain ambiguous, but TPO-antibodies in the third trimester may be a predictor of late PPD.

## Introduction and background

Postpartum mood disorders (PMDs) are currently a leading cause of maternal morbidity and mortality in relation to pregnancy. Furthermore, there is an associated increased risk of impaired maternal-infant attachment and parenting behaviors, and risk of infanticide [1]. Women are at the highest risk of mood disorders around pregnancy, with 1/7 of women being treated for depression within one year before and after delivery [2]. There has been a recent push for increased attention towards maternal follow-up and care in the postpartum period, to the point where it may be referred to as “the fourth trimester” to emphasize its importance [3]. PMD include postpartum blues (PB), postpartum depression (PPD) (and/or anxiety), and postpartum psychosis [3]. The significant morbidity and mortality attributed to PMDs are obvious, yet an inadequate understanding of pathophysiology in the process of prevention and development of antenatal screening guidelines. Currently, screening is limited to postpartum assessments and no reliable antepartum predictors have been identified to aid in early screening [1,2].

The search for a biomarker of PMD has been elusive, yet thyroid markers remain appealing due to the association between thyroid dysfunction and major depression [4]. The thyroid hormone pathways are significantly involved in numerous organ systems, including the reproductive system. Meta-analyses support a persistent correlation between clinical depression hypothyroidism and thyroid autoimmunity [5,6]. However, the exact relationship between thyroid function and PMD remains unclear.

Therefore, this study aimed to understand how thyroid endocrine function may be related to PMDs and whether it may be utilized as a predictor of PMDs. Ultimately, the goal was to understand better the role of thyroid function in the pathophysiology of PMDs, and to allow for better preventative guidance in the prenatal period for suspected high-risk patients. 

## Review

Methodology

The study was designed as a narrative review of past and current literature on thyroid hormones, auto-antibodies, and function in relation to PMD. Several database searches, and subsequent reviews of pertinent citations, were conducted to identify studies that met inclusion criteria. In-text citations of studies were further examined if they reported primary data outcomes pertinent to the inclusion criteria of this review. Studies were included if they discussed any marker related to thyroid endocrinology in relation to the incidence or pathophysiology of any PMDs. Both primary and secondary studies were included in the initial database searches. The permissive inclusion criteria were used due to the relative scarcity of research on the topic and the ambiguous pathophysiology of PMD. Articles were excluded if they were not open-access did not report outcomes related to mood disorders during the postpartum period, did not mention thyroid function or hormones, or did not include human outcomes. Although it was noted that different postpartum affective disorders have varying pathophysiology, the full spectrum was researched to allow for potential comparisons of similarities and contrasts.

**Figure 1:**
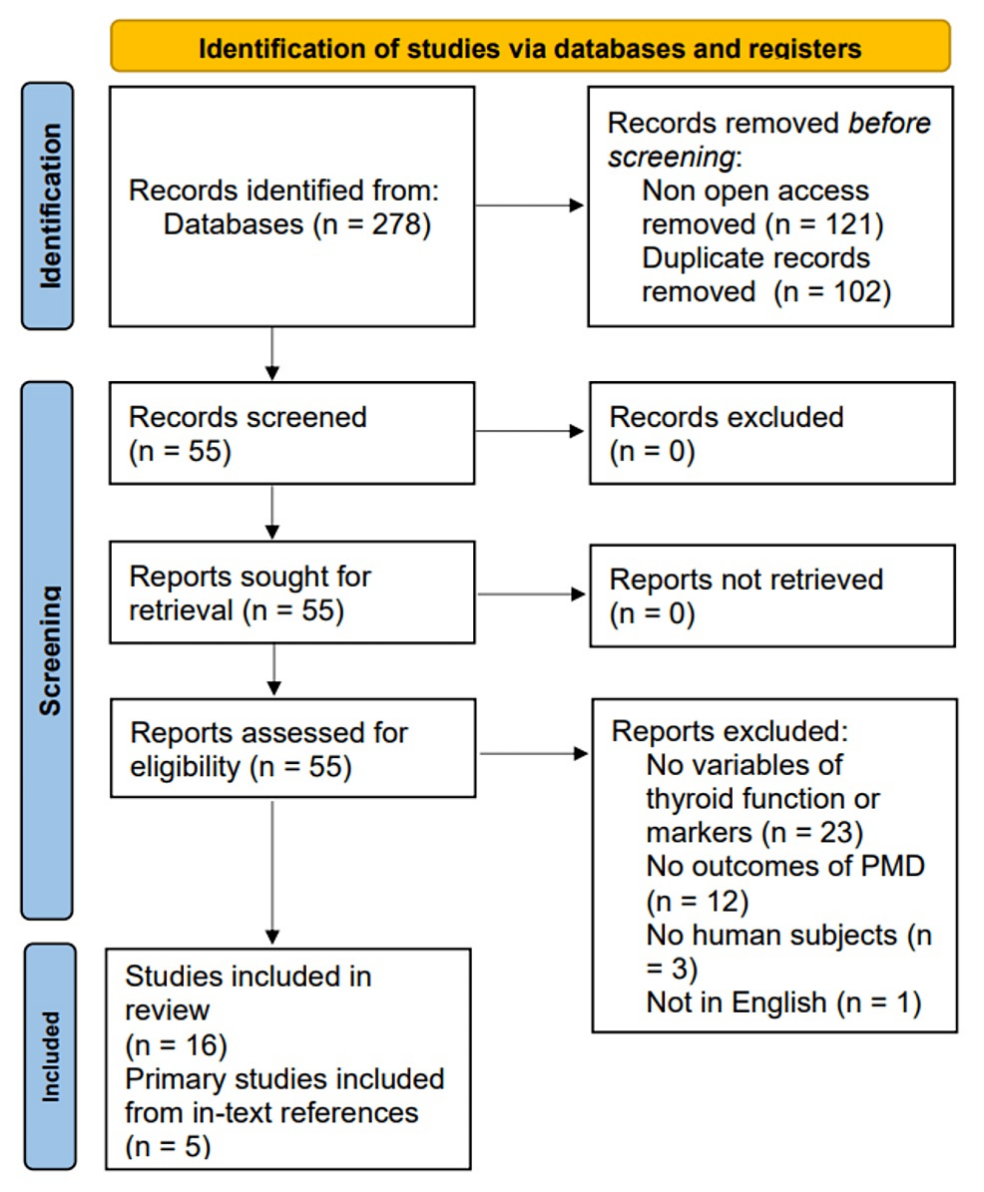
Flow diagram summarizing the literature screening process; based on the PRISMA 2020 template PRISMA: Preferred Reporting Items for Systematic Reviews and Meta-Analyses

PubMed was utilized as the primary database for literature searches, with six searches performed. Search terms included “postpartum mood disorders AND thyroid OR hypothyroid OR thyroid dysfunction OR endocrinology OR iodine deficiency” and “autoimmune thyroid postpartum depression”. Only open-access, full-text, peer-reviewed articles were obtained, which yielded a total of 278 results. Duplicates and articles not open access were removed, which excluded 102 and 121 results, respectively. The remaining 55 results were reviewed based on the inclusion and exclusion criteria. The articles were further narrowed down to a total of 16 that were included in the final references. Another five articles reporting pertinent primary research were obtained from in-text citations of those articles. An additional 18 peer-reviewed articles were included for foundational background information and definitions. A total of 39 peer-reviewed articles were ultimately included based on the above methodology. The protocol highlighted a scarcity of research on PB, anxiety, and psychosis, as most articles examined PPD. Thus, the scope of this study was narrowed and limited in terms of the spectrum of PMD. A Preferred Reporting Items for Systematic Reviews and Meta-Analyses (PRISMA) 2020 template was utilized to create a flow chart illustrating the literature search process, which can be seen in Figure 1 below.

Postpartum mood disorders

Definition of PMDs

Perinatal mental illness and PMDs are blanket terms for a myriad of psychiatric disorders ranging from mild depression, anxiety, mania, or psychosis, which can occur prior to or during pregnancy, or postpartum up to one year after delivery [7]. The most common medical complication of childbearing is perinatal depression (PND) or PPD [8]. PND is sometimes used interchangeably with PPD; both typically refer to major or minor depression that begins during pregnancy or up to 12 months following childbirth [9]. PPD can be defined symptomatically as exceeding a threshold on a screening measure such as the Edinburgh Postnatal Depression Scale (EPDS) or more rigorously in the context of the Structured Clinical Interview for Diagnostic and Statistical Manual of Mental Disorders (DSM)-IV [7].

The DSM-IV defines an episode of major depression after delivery as two weeks or more of persistent depressed mood, or loss of interest in daily activities, along with four associated symptoms (appetite disturbance, sleep disturbance, psychomotor agitation or slowing, fatigue, feelings of worthlessness or inappropriate guilt, poor concentration, suicidal ideation), which occur within four weeks following childbirth [8]. Of note, there are several notable issues in defining PPD such as a consensus in the time frame that is considered the “postpartum” period, along with distinguishing whether PPD is a distinct entity from MDD [9].

Additionally, it is often difficult to distinguish whether depressive symptoms are distinct from the challenges of infant care in a “normal” postpartum experience which leads to fatigue, disrupted sleep, and loss of appetite, all of which can easily be mistaken for symptoms of depression. Maternity blues (MB), also known as the baby blues, PB, or postnatal blues, is different from PPD due to its transient nature of mild, self-limited depressive symptoms [10]. The symptoms of MB include brief crying spells, irritability, nervousness, poor sleep, and emotional reactivity beginning one-two days after childbirth and resolving within 10 days [8]. Despite its transient nature, identification of MB is of utmost importance, as it is a well-established risk factor for more severe PMDs, such as PPD and postpartum psychosis [10].

Postpartum psychosis can be defined as a severe form of major depression along with the onset or recurrence of a primary psychotic disorder [11]. The onset of symptoms is rapid, usually within the first one-four weeks after childbirth, or as early as two-three days. Symptoms include frank psychosis, delusions (paranoid, grandiose, or bizarre), fluctuations in mood, confusion, and grossly disorganized behavior. The symptoms of postpartum psychosis have been noted to differ from psychosis that is unrelated to childbearing, and although rare, maternal psychotic symptoms can include altruistic homicidal delusions, where the mother believes that in killing her child, she is saving them from a fate that is worse than death [7]. As such, postpartum psychosis is considered a psychiatric emergency, and prompt diagnosis is critical to ensure the safety of both the mother and her child. An important diagnostic clue for postpartum psychosis is a mother with pre-existing bipolar disorder, and current data suggests that postpartum psychosis is actually an overt presentation of bipolar disorder that coincides with the massive hormonal changes following delivery [7,11]. Notably, undiagnosed bipolar disorder has been found in half of women with "treatment resistant" PPD [7,12].

Diagnosis of PMDs

It is established that the use of universal depression screening in the postpartum period significantly improves detection rates and is therefore an essential component of postpartum care [8,13]. The most widely utilized screening tool for PPD is the EDPS, which employs self-reporting of 10 items on a scale of 0-3 [8,13]. A score of 13 or 15 and above, with or without a history of depressive disorders, respectively, is considered the cutoff for a positive result [8]. Alternatively, the EDPS-P (partner) screening uses the responses of the significant other to assess the mother’s depressive symptoms and has been evaluated as a reliable option to decrease self-reporting bias [8]. However, there are several other acceptable screening tools for PPD, such as the Beck Depression Inventory (BDI), Hamilton Rating Scale for Depression (HSRD), Patient Health Questionnaire (PHQ-9), Postpartum Depression Screening Scale (PDSS), and Center for Epidemiologic Studies of Depression instrument (CES-D) [8]. Screening should be employed starting at the first prenatal visit [8]. There are, however, a few caveats related to postpartum screening tools. Comfort and acceptability of participation have been negatively correlated with depression risk, and thus high-risk patients are the least likely to follow up and undergo screening. Additional obstacles to screening can include underemployment of screening tools by providers, cultural variations in symptom manifestation, and language barriers in minority populations [8,14]. Furthermore, as onset may occur as late as a month postpartum, PPD may go undetected in mothers who are lost to follow-up after the early neonatal period.

The diagnosis of other PMD conditions is less clear. PB may be diagnosed clinically, as it has milder symptoms, does not meet the cut-off of the EDPS, and typically manifests earlier than PPD, with onset one-two days postpartum and resolution within 10 days [10]. Similarly, postpartum psychosis is also diagnosed clinically with symptoms consistent with an acute psychotic episode in the postpartum period but lacks a widely accepted screening tool [3].

The value of further predictive markers of PPD is emphasized, due to the lack of standardized screening tools for PB and postpartum psychosis and the potential fallacies of self-reported screening tools in the postpartum period. Potential endocrine (i.e., thyroid hormones) markers that can be clinically measured in the prenatal period may provide improved clinical suspicion of PMD and ensure subsequent preventative measures for the postpartum period.

Thyroid Endocrinology and PMDs

Thyroid function and psychiatric symptoms have been consistently linked and thyroid dysregulation is pervasive in pregnancy, thus demanding exploration of a possible relationship [4,5,15]. Indeed, thyroid dysregulation may affect anywhere between 7-23% of women in the postpartum period (with variations between sources), relative to 3-4% in general populations [15,16]. The relationship between thyroid hormones and mood symptoms may be attributed to their effects on neurotransmitters such as serotonin, norepinephrine, and dopamine [2]. A relationship between thyroid hormone and postsynaptic beta-adrenergic receptors has been suggested and may relate lower functional catecholamine activity with low thyroxine (T4) levels [2]. Similarly, low serotonergic activity in the CNS has been associated with low thyroid hormone activity, further implying a pathophysiological relationship considering the central role of serotonin and catecholamines in mood disorders [17]. The relationship may be further complicated as transthyretin (TTR), which transports T4 across the blood-brain barrier (BBB), has been demonstrated to decrease activity in depressed patients. Thereby, decreased TTR levels could predispose patients to decreased stimulation of T4 on catecholamine and serotonergic CNS activity in low T4 states. Although this theory may partially illustrate a relationship, it does not explain other possible predisposing factors during the prenatal to the postnatal period, as mood disorders are significantly more likely during this period [2].

There is strong evidence to support elevated levels of thyroid-binding globulin (TBG) during pregnancy because of increased hepatic production during the concurrent hyperestrogenic state [18]. As TBG initially decreases free T4 levels by binding serum T4, it may be hypothesized that less T4 is available to cross the BBB to exert stimulatory effects on serotonergic and catecholaminergic activity in the CNS. In fact, serum TBG levels have been found as a predictor of PPD [18]. Furthermore, lower baseline levels of TTR in patients with comorbid mood disorders may exacerbate their vulnerability to decreased serum free T4 levels in pregnancy, as less T4 is available to enter the CNS with baseline impairment of transport across the BBB. Nevertheless, the theory is strictly hypothetical and no primary research on the relationship between TTR, T4, and PMD incidence has been conducted to our knowledge.

Despite extensive research on cortisol and corticortopin-releasing hormone (CRH) as components of PMD pathophysiology, they have not been demonstrated as consistent markers of incidence [1]. However, they may be associated with thyroid dysfunction. CRH is produced by the placenta in a positive-feedback loop with endogenous cortisol, which leads to a peak cortisol level two to five times nonpregnant baseline (comparable to levels in severe hypothalamic-pituitary-adrenal (HPA) axis dysregulation), in the third trimester [1,15,19,20]. Upon delivery of the placenta, there is a rapid drop in cortisol and a progressive increase of hypothalamic CRH production in response [1,19]. In addition, hyporesponsiveness of HPA-axis may be persistent post-parturition and imply continued endocrine dysregulation [15]. Clinical studies have demonstrated relationships between elevated CRH and PPD at four and six weeks postpartum [21,22]. Another study correlated elevated third-trimester CRH with PPD symptoms at late postpartum time points of 12 weeks and a year postpartum [23]. Considering the immunosuppressive attributes of cortisol, its elevation may be protective against thyroid autoimmunity during pregnancy [20]. The rapid drop in cortisol postpartum may therefore allow a flare-up of thyroid autoimmunity and dysregulation during the eventual postpartum period. Autoimmune thyroiditis and hypothyroidism have a demonstrated correlation with clinical depression based on meta-analyses [5,6]. Subclinical thyroiditis in the postpartum period, or postpartum thyroiditis, often produces a late hypothyroid phase after stored T4 has been depleted, which may correlate with depressive symptoms in the late postpartum period [24,25].

Thyroid Hormones as Markers of PMDs

Thyroid hormones demonstrate both promise and difficulty as predictive markers of PMD. Thyroid dysfunction can predispose to major depressive disorder (MDD), but thyroid hormones also fluctuate significantly with the course of pregnancy [1,4]. Nevertheless, there appears to be a consistent correlation between hypothyroidism, subclinical hypothyroidism, and PMDs. The relationship is most notable with postpartum depressive disorders [26].

A study of 227 women revealed that thyroid-stimulating hormone (TSH) levels above 4.0 mIU/L (during labor) were significantly associated with an increased risk of PPD (EDPS above 12) at six months postpartum [27]. However, replication is necessary due to the small sample size, with only 26 women being diagnosed with PPD. Furthermore, obtaining EDPS scores earlier than six months may also provide further evidence of a possible correlation. Additionally, there are other studies that fail to associate varying peripartum TSH levels with PPD, thus raising considerable doubt regarding its use as a marker [28,29]. When considering clinical application, it is also important to consider the logistical difficulties of obtaining serum TSH levels during the intense process of labor. Nevertheless, the outcomes are promising and may be an indicator of a relationship between a perinatal hypothyroid state and the incidence of PPD.

A 2007 prospective cohort study of euthyroid pregnant women by Pedersen et al. obtained weekly serum thyroid hormone levels during weeks 32-37 of the third trimester and weekly PPD screening (EPDS and BDI) scores 2-24 weeks postpartum [26]. The outcomes revealed a significant, negative correlation between total and free T4 in the third trimester and PPD screening scores. The researchers concluded that third-trimester total/free T4 levels in the low euthyroid range may significantly correlate with positive PPD scores postpartum. Unsurprisingly, the correlation was strongest in women with a history of major or minor depression. There was a less significant relationship observed with triiodothyronine (T3) levels [26]. Another prospective cohort of 93 women mirrored these results with a significant negative correlation between free T4 and subjective depressive symptoms in the second and third trimesters [30]. The study also determined a positive correlation between reverse triiodothyronine (rT3) and depressive symptoms postpartum, although the study only extended one week postpartum [30].

Similarly, a recent multicenter RCT revealed a depression incidence of more than 1/4 among pregnant women with subclinical hypothyroidism (elevated TSH and T4/T3 in the normal or low normal range) [2]. Despite the high incidence of depression among pregnant women with subclinical hypothyroidism, it appears that proactive screening and treatment did not improve outcomes when comparing T4 and placebo for the primary outcome of postnatal depressive symptoms [2].

Interestingly, a large Norwegian study found a correlation between iodine deficiency and depressive symptoms (measured by EPDS) both perinatally and postnatally [31]. The researchers inferred that the relationship could be explained by the increased demands and thyroid hormone production during this time, and the failure to meet these demands in iodine-deficient individuals [31]. Nevertheless, the outcomes further emphasize the role of thyroid function in PPD.

Thyroid hormone levels in the antenatal period appear to have a relationship with the etiology of PPD, yet the exact relationship remains unclear and certain markers like TSH lack evidence. Unfortunately, using thyroid hormones as predictive markers of PMD remains difficult at this time as previous studies have failed to improve PPD outcomes with proactive T4 screening, the hormone levels fluctuate significantly, and there is no consistent evidence to suggest a mechanism to explain how lower T4 levels antenatally may explain PPD [2].

Thyroid Autoantibodies as Markers of PMDs

Thyroid autoimmunity during the antenatal period has been consistently correlated with increased risk of PPD and postpartum depressive symptoms [15,16,20,25,32-34]. Specifically, recent systematic reviews and meta-analyses highlight a significantly increased risk of postpartum depressive symptoms with the presence of anti-TPO antibodies; one 2020 meta-analysis of 2,932 subjects showcased a relative risk of 1.41 (confidence interval (CI) 1.11-2.0, p = 0.008) [16,34,35].

A prospective cohort of 1,075 women in the 2018 study by Wesseloo et al. showed that positive anti-TPO antibody status had a 3.8 odds ratio (OR) (95% CI 1.3-11.6) of PPD at four months by EPDS diagnosis [33]. Although the study highlights promising results, a significant correlation was not observed at other postpartum time points. Similarly, a 2016 study demonstrated a positive correlation between TPO autoantibodies and PPD symptoms at six months postpartum [32].

**Figure 2:**
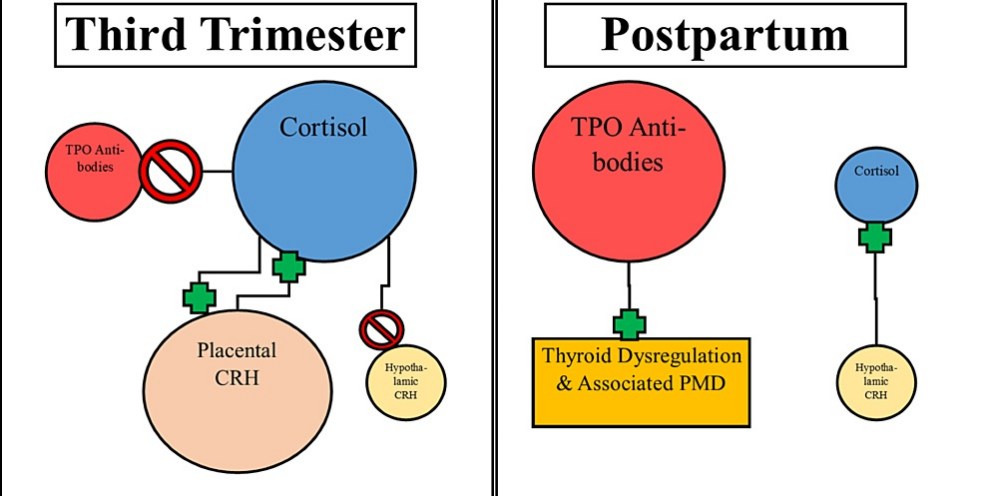
Visual illustration of alterations in cortisol and thyroid autoimmunity before and after parturition. The left side of the figure demonstrates the positive feedback loop between placental corticotropin-releasing hormone (CRH) and cortisol, which exhibits suppressive effects on anti-thyroid peroxidase (TPO) antibodies and hypothalamic CRH. Following delivery of the placenta, cortisol levels decrease rapidly, with insensitive and insufficient stimulation by hypothalamic CRH, leaving minimal immunosuppressive action on thyroid autoantibodies and subsequent clinical predisposition to associated postpartum mood disorders (PMD). Figure 2 was made by one of the authors of this paper.

A double-blinded prospective cohort study, published in BMJ in 1992, also demonstrated a significant positive correlation between thyroid autoantibodies (regardless of thyroid state) and depressive symptoms at all time points between six and 28 weeks postpartum [20]. Notably, there was no significant correlation with anxiety symptoms, or major depression (by DSM criteria), and no incidence of postpartum psychosis was observed [20]. The authors hypothesized a possible explanation of the findings in relation to cortisol. The elevated cortisol levels during pregnancy may have suppressed thyroid autoimmunity, with a subsequent flare-up of autoimmunity and symptoms with a rapid decrease of cortisol postpartum; the symptomatic discomfort of thyroid dysregulation may have accounted for depressive symptoms [20]. Similar conclusions were proposed from the 2013 study by Groer et al., which also supported a flare-up of autoimmunity and dysphoric symptoms postpartum in women with positive TPO antibody status [32]. In fact, a 2018 study by Sakkas et al. measured serum titers of TPO antibodies and serum cortisol in the third trimester and postpartum, which revealed a significant increase of TPO antibodies at one week postpartum and a significant negative correlation between anti-thyroglobulin (TG) antibodies in the first week postpartum (r = -0.419, p < 0.05) [36]. The study concluded that cortisol, in addition to estrogen and transforming growth factor-beta 1 (TGF-β1), was associated with suppression of thyroid autoimmunity in the pregnancy and postpartum period [36]. The proposed relationship between cortisol fluctuation and thyroid autoimmunity before and after parturition is illustrated in Figure 2 below.

The correlation between mild to moderate PPD and TPO antibodies has been consistently observed in women, regardless of the incidence of postpartum thyroiditis [16,20,25,34]. The studies also hint at a common trend of late postpartum depressive symptoms [16,20,32,33]. The late onset may be partially explained by the late hypothyroid phase of postpartum thyroiditis (even if subclinical), as hypothyroidism has been independently associated with clinical depression [5,16].

It appears that thyroid autoimmunity may have a pathophysiological relationship to PPD. However, there is insufficient evidence to suggest a relationship to the incidence of PB or psychosis as studies have not examined the early postpartum period (timeline for PB) and lacked assessment of psychotic symptoms respectively. To note, an isolated case report described significant elevations of anti-TG and anti-TPO antibodies in a young female with severe postpartum psychosis [37]. Nevertheless, presence of TPO autoantibodies may be a predictor of PPD or subclinical depressive symptoms [20,32,33]. As the endogenous corticosteroid cortisol exhibits immunosuppressive qualities, its rapid decrease in the postpartum period may account for a flare-up of thyroid symptoms in TPO-positive women [20].

In clinical practice, the use of TPO antibodies as a predictor of PPD seems promising but demands tailored research. Up to 50% of women with anti-TPO antibodies in early pregnancy will develop postpartum thyroiditis, making it a useful marker in identifying those at risk [38,39]. Other studies indicate that as many as 80% of women with positive TPO antibodies in the third trimester may subsequently develop postpartum thyroiditis [24]. In these women, as well as women with known autoimmune disease, thyroid function tests are indicated at three and six months postpartum, and further investigation can help determine whether there is a correlation between TPO antibodies and PPD. Theoretically, implementation of late antenatal (i.e. third trimester) TPO antibody screening could be useful to identify high-risk patients as targets for frequent postpartum EDPS screening and preventative education and counseling on PPD. Ultimately, a clinical study would be needed to examine if TPO antibody screening can significantly improve postpartum follow-up and EDPS screening, PPD outcomes, adherence to SSRI or CBD treatment, or other pertinent outcomes.

Discussion

The objective of this narrative literature review was to explore the relationship between thyroid endocrine function and PMD and investigate whether thyroid markers could be utilized as predictors of PMD. Thyroid dysfunction, such as hypothyroidism or thyroid autoimmunity, has already been noted to play a role in MDDs. However, the link between thyroid dysfunction and PMDs specifically is still unclear. Based on articles that matched our search criteria, we were able to explore different possible predictors of PPD including TSH, TTR, TBG, and TPO antibodies that showed a positive relationship in one way or another.

Several studies illustrated the association between thyroid hormones and PMD. Elevated levels of TSH during labor are significantly associated with an increased risk of PPD at 6 months postpartum [27], however, other studies cast doubt on this association [28,29]. Low levels of total and free T4 in the third trimester were also correlated with PPD in a prospective cohort study conducted by Pedersen et al [26]. Moreover, even subclinical hypothyroidism during pregnancy has been associated with a higher incidence of depression in the postpartum period [2]. Nevertheless, proactive screening and treatment with T4 for subclinical hypothyroidism during pregnancy did not improve outcomes of postnatal depressive symptoms, inferring further complexity of the pathophysiologic interplay [2].

The relationship between TPO antibodies and PPD was most notable. Multiple studies found a significant, positive correlation between PPD symptoms and TPO antibodies. Wesseloo et al. found that the presence of TPO antibodies had an OR of 3.8 (95% CI 1.3-11.6) (relative to the absence of the autoantibodies) for developing PPD at four months postpartum, while Groer and Vaughan found a similar significant positive correlation of TPO antibodies and PPD at six months postpartum [32,33]. TPO antibodies seem to be a promising marker to continue further exploring their role in PMD. Part of this promise may be attributed to the interplay with cortisol. As cortisol levels peak and subsequently drop in the postpartum period, the loss of its immunosuppressive effects may enable a bout of hypothyroid autoimmunity, and associated depressive symptoms, in TPO antibody-positive mothers [20,36]. A 2018 study demonstrated the negative correlation between cortisol and thyroid autoantibodies postpartum, and their peaks and nadirs, respectively, at 36 weeks of pregnancy [36]. Despite the associations between cortisol, TPO antibodies, and PPD, the relationship is still likely multifactorial, and further studies are needed to determine the causation and clinical utility of TPO antibodies as an isolated marker for PPD.

A large limitation to studying this relationship is the complex pathophysiology of PMD that is yet to be fully understood. The physiological changes throughout pregnancy are dictated by many interconnected processes that may each impact and predispose women to changes in the postpartum period leading to PMD. The fluctuating nature of thyroid hormones during pregnancy and postpartum, along with the lack of consistent evidence on causative mechanisms linking thyroid function and PPD, complicate their clinical application. This further poses a challenge to isolate and explore a specific relationship, such as TPO antibodies and PMD. An additional limitation is the population being studied. Pregnant individuals are a vulnerable population and clinical studies can be difficult to challenging and implement during antenatal and postpartum periods.

Looking into the future, more studies are needed to explore this relationship as there was a limited number of studies due to the scarcity of available research on this topic. Future studies need to include larger sample sizes, as the sample sizes in the articles explored were relatively small and overall limited the generalizability of the findings. A promising marker for PPD related to thyroid dysfunction is TPO antibodies, and research should be tailored for clinical studies to explore the intricate endocrine mechanisms involving both thyroid function and mental health. Identifying reliable predictors of PMD may lead to better preventive guidance and targeted interventions during the prenatal period for high-risk patients, ultimately improving maternal and infant health outcomes.

## Conclusions

Thyroid dysfunction may be related to the pathophysiology of PPD via a transient hypothyroid state in the late postpartum period. Women with TPO antibodies in the third trimester may be asymptomatic, due to immunosuppressive effects of elevated cortisol. However, following the precipitous drop of cortisol postpartum, autoimmune thyroiditis may deplete T4 storages leading to a hypothyroid and depressive state in the late postpartum period. TPO antibody positivity is associated with an increased risk of PPD symptoms past the PB timeline. Antenatal TPO antibody titers may be helpful to identify patients at high risk for PPD. Still, an elaboration of pathophysiology and clinical studies is necessary to determine if the implementation of this strategy can lead to significant improvement in PPD outcomes. Although thyroid autoimmunity may be a helpful predictor of PPD, it may not account for all cases of PPD, as the etiology is likely multifactorial and demands extensive further research.

## References

[1] Guintivano J, Manuck T, Meltzer-Brody S (2018). Predictors of postpartum depression: a comprehensive review of the last decade of evidence. Clin Obstet Gynecol.

[2] Costantine MM, Smith K, Thom EA (2020). Effect of thyroxine therapy on depressive symptoms among women with subclinical hypothyroidism. Obstet Gynecol.

[3] Osborne LM (2018). Recognizing and managing postpartum psychosis: a clinical guide for obstetric providers. Obstet Gynecol Clin North Am.

[4] Schiller CE, Meltzer-Brody S, Rubinow DR (2015). The role of reproductive hormones in postpartum depression. CNS Spectr.

[5] Bode H, Ivens B, Bschor T, Schwarzer G, Henssler J, Baethge C (2021). Association of hypothyroidism and clinical depression: a systematic review and meta-analysis. JAMA Psychiatry.

[6] Siegmann EM, Müller HH, Luecke C, Philipsen A, Kornhuber J, Grömer TW (2018). Association of depression and anxiety disorders with autoimmune thyroiditis: a systematic review and meta-analysis. JAMA Psychiatry.

[7] O'Hara MW, Wisner KL (2014). Perinatal mental illness: definition, description and aetiology. Best Pract Res Clin Obstet Gynaecol.

[8] Sit DK, Wisner KL (2009). Identification of postpartum depression. Clin Obstet Gynecol.

[9] Batt MM, Duffy KA, Novick AM, Metcalf CA, Epperson CN (2020). Is postpartum depression different from depression occurring outside of the perinatal period? A review of the evidence. Focus (Am Psychiatr Publ).

[10] Tosto V, Ceccobelli M, Lucarini E, Tortorella A, Gerli S, Parazzini F, Favilli A (2023). Maternity blues: a narrative review. J Pers Med.

[11] Sit D, Rothschild AJ, Wisner KL (2006). A review of postpartum psychosis. J Womens Health (Larchmt).

[12] Sharma V, Burt VK, Ritchie HL (2009). Bipolar II postpartum depression: Detection, diagnosis, and treatment. Am J Psychiatry.

[13] Evins GG, Theofrastous JP, Galvin SL (2000). Postpartum depression: a comparison of screening and routine clinical evaluation. Am J Obstet Gynecol.

[14] Di Florio A, Putnam K, Altemus M (2017). The impact of education, country, race and ethnicity on the self-report of postpartum depression using the Edinburgh Postnatal Depression Scale. Psychol Med.

[15] Skalkidou A, Hellgren C, Comasco E, Sylvén S, Sundström Poromaa I (2012). Biological aspects of postpartum depression. Womens Health (Lond).

[16] Le Donne M, Mento C, Settineri S, Antonelli A, Benvenga S (2017). Postpartum mood disorders and thyroid autoimmunity. Front Endocrinol (Lausanne).

[17] Cleare AJ, McGregor A, O'Keane V (1995). Neuroendocrine evidence for an association between hypothyroidism, reduced central 5-HT activity and depression. Clin Endocrinol (Oxf).

[18] Pedersen C, Leserman J, Garcia N, Stansbury M, Meltzer-Brody S, Johnson J (2016). Late pregnancy thyroid-binding globulin predicts perinatal depression. Psychoneuroendocrinology.

[19] Dickens MJ, Pawluski JL (2018). The HPA axis during the perinatal period: implications for perinatal depression. Endocrinology.

[20] Harris B, Othman S, Davies JA (1992). Association between postpartum thyroid dysfunction and thyroid antibodies and depression. BMJ.

[21] Yim IS, Glynn LM, Dunkel-Schetter C, Hobel CJ, Chicz-DeMet A, Sandman CA (2009). Risk of postpartum depressive symptoms with elevated corticotropin-releasing hormone in human pregnancy. Arch Gen Psychiatry.

[22] Iliadis SI, Sylvén S, Hellgren C (2016). Mid-pregnancy corticotropin-releasing hormone levels in association with postpartum depressive symptoms. Depress Anxiety.

[23] Hahn-Holbrook J, Schetter CD, Arora C, Hobel CJ (2013). Placental corticotropin-releasing hormone mediates the association between prenatal social support and postpartum depression. Clin Psychol Sci.

[24] Prummel MF, Wiersinga WM (2005). Thyroid peroxidase autoantibodies in euthyroid subjects. Best Pract Res Clin Endocrinol Metab.

[25] Lazarus JH, Ammari F, Oretti R, Parkes AB, Richards CJ, Harris B (1997). Clinical aspects of recurrent postpartum thyroiditis. Br J Gen Pract.

[26] Pedersen CA, Johnson JL, Silva S, Bunevicius R, Meltzer-Brody S, Hamer RM, Leserman J (2007). Antenatal thyroid correlates of postpartum depression. Psychoneuroendocrinology.

[27] Sylvén SM, Elenis E, Michelakos T, Larsson A, Olovsson M, Poromaa IS, Skalkidou A (2013). Thyroid function tests at delivery and risk for postpartum depressive symptoms. Psychoneuroendocrinology.

[28] Szpunar MJ, Parry BL (2018). A systematic review of cortisol, thyroid-stimulating hormone, and prolactin in peripartum women with major depression. Arch Womens Ment Health.

[29] Noshiro K, Umazume T, Inubashiri M, Tamura M, Hosaka M, Watari H (2023). Association between Edinburgh Postnatal Depression Scale and serum levels of ketone bodies and Vitamin D, thyroid function, and iron metabolism. Nutrients.

[30] Konstantakou P, Chalarakis N, Valsamakis G (2021). Associations of thyroid hormones profile during normal pregnancy and postpartum with anxiety, depression, and obsessive/compulsive disorder scores in euthyroid women. Front Neurosci.

[31] Brantsæter AL, Garthus-Niegel S, Brandlistuen RE, Caspersen IH, Meltzer HM, Abel MH (2022). Mild-to-moderate iodine deficiency and symptoms of emotional distress and depression in pregnancy and six months postpartum - Results from a large pregnancy cohort. J Affect Disord.

[32] Groer MW, Vaughan JH (2013). Positive thyroid peroxidase antibody titer is associated with dysphoric moods during pregnancy and postpartum. J Obstet Gynecol Neonatal Nurs.

[33] Wesseloo R, Kamperman AM, Bergink V, Pop VJ (2018). Thyroid peroxidase antibodies during early gestation and the subsequent risk of first-onset postpartum depression: a prospective cohort study. J Affect Disord.

[34] Minaldi E, D'Andrea S, Castellini C, Martorella A, Francavilla F, Francavilla S, Barbonetti A (2020). Thyroid autoimmunity and risk of post-partum depression: a systematic review and meta-analysis of longitudinal studies. J Endocrinol Invest.

[35] Dama M, Steiner M, Lieshout RV (2016). Thyroid peroxidase autoantibodies and perinatal depression risk: a systematic review. J Affect Disord.

[36] Sakkas EG, Paltoglou G, Linardi A (2018). Associations of maternal oestradiol, cortisol, and TGF-β1 plasma concentrations with thyroid autoantibodies during pregnancy and postpartum. Clin Endocrinol (Oxf).

[37] Lalanne L, Meriot ME, Ruppert E, Zimmermann MA, Danion JM, Vidailhet P (2016). Attempted infanticide and suicide inaugurating catatonia associated with Hashimoto's encephalopathy: a case report. BMC Psychiatry.

[38] Smith A, Eccles-Smith J, D'Emden M, Lust K (2017). Thyroid disorders in pregnancy and postpartum. Aust Prescr.

[39] Stagnaro-Green A (2012). Approach to the patient with postpartum thyroiditis. J Clin Endocrinol Metab.

